# Role of Extended White Blood Cell Parameters in Distinguishing Acute Febrile Illnesses

**DOI:** 10.1155/ah/8080147

**Published:** 2025-04-21

**Authors:** Tandry Meriyanti, Maroloan Aruan, Glorya N. D. Ananda

**Affiliations:** ^1^Medical Laboratory Technology Department, Faculty of Health Science, Pelita Harapan University, Tangerang, Banten, Indonesia; ^2^Laboratory Department, Siloam Hospitals Lippo Village, Tangerang, Banten, Indonesia

## Abstract

**Introduction:** Acute febrile illness contributes to significant morbidity and death particularly in tropical country such as Indonesia. The symptoms are nonspecific, therefore distinguishing these pathogens is difficult without additional laboratory tests. The extended white blood cell parameters indicate cell activities induced by immune response to infection. The study aims to explore the profile of extended white blood cell parameters in acute febrile illnesses and evaluate their diagnostic power to differentiate etiologies of acute febrile illnesses.

**Methods:** This study was a cross-sectional analytical study with a total of 473 samples, conducted between October 2022 and 2023 at Siloam Hospitals Lippo Village, Banten, Indonesia. Acute febrile illnesses are included in this study, including dengue infection, chikungunya infection, typhoid infection, and other bacterial infections. The extended white blood cell parameters including high fluorescence lymphocyte count (HFLC), immature granulocyte (IG), neutrophil-to-lymphocyte ratio (NLR), and cell population data (CPD) which were NE-SSC, NE-SFL, NE-WY, LY-X, LY-Y, and LY-WY. These parameters were integrated in a routine hematology test as research parameters, performed by Sysmex XN2000. Data were analyzed using SPSS Version 25.

**Results:** The value of extended white blood cell parameters was found to be significantly different in viral and bacterial infection (HFLC 1.10% (0.30%–3.85%) vs. 0.20% (0.10%–0.70%), *p* < 0.001; IG 0.4% (0.2%–0.6%) vs. 0.5% (0.3%–1.1%), *p* < 0.001; NLR 1.93 (1.10–3.47) vs. 5.21 (2.20–12.26), *p* < 0.001; NE-SFL 47.7 (45.95–50.10) vs. 48.6 (45.82–52.57), *p*=0.020; NE-WY 622 (585–653) vs. 653 (615–747), *p* < 0.001; LY-Y 66.4 (63.85–69.75) vs. 64.05 (60.52–67.17), *p* < 0.001). HFLC and LY-Y had statistically significant AUC 0.753 and 0.646, respectively, (*p* < 0.001) in the dengue infection group. IG, NLR, NE-WY, and NE-SFL had statistically significant AUC in bacteremia (0.806, 0.876, 0.783, and 0.656, respectively).

**Conclusion:** HFLC was a useful diagnostic tool to identify viral infection, particularly dengue infection, while IG, NLR, NE-SFL, and NE-WY can be useful to differentiate bacteremia from other acute febrile illnesses.

## 1. Introduction

Fever is one of the most common symptoms for patients to have medical treatment in hospitals. Acute febrile illness contributes to significant morbidity and death among children and adults, particularly in tropical countries such as Indonesia. Viruses and bacteria are the main causes of acute febrile illness, including dengue virus, chikungunya virus, Salmonella, and other bacterial infections. The symptoms are nonspecific and similar; therefore, distinguishing these pathogens is difficult without additional laboratory testing. On the other hand, accurate and early diagnosis is important to prevent inappropriate treatment, particularly antibiotic prescribing. Confirmatory laboratory tests, including microbiology culture, molecular serology, antigen–antibody rapid test, and biomarker tests for assessment of immune response such as C-reactive protein or procalcitonin, can be useful to help distinguish the pathogens, but the availability of the test is limited and requires additional cost [1].

Hematology tests are routinely ordered for patients with fever and may be helpful since leukopenia and thrombocytopenia are specific hematology changes of dengue infection, but these parameters are not specific for other febrile illnesses [1, 2]. In general, leukocytosis, absolute neutrophil count, percentage of neutrophils, and immature granulocytes (IGs) have been used to represent bacterial infection, while lymphocytosis and atypical lymphocytes are usually found in viral infection, but these parameters have low sensitivity and specificity for distinguishing acute febrile illnesses [3, 4].

Automated hematology analyzers use the fluorescence flow cytometry method to examine the cell membrane composition and cytoplasmic activity of white blood cell parameters; hence, neutrophils and lymphocytes can be classified more specifically based on cell activities induced by the immune response to infection. These extended white blood cell parameters, including high fluorescence lymphocyte count (HFLC), IGs, neutrophil-to-lymphocyte ratio (NLR), and cell population data (CPD), are integrated into routine hematology tests [3–5]. Previous studies showed that higher IG and NLR are associated with bacterial infection [6–9], while HFLC is associated with viral infection [10, 11]. Among CPD, neutrophil cell complexity (NE-SSC), neutrophil fluorescence intensity (NE-SFL), and neutrophil fluorescence distribution width index (NE-WY) have been reported as potential biomarkers for acute bacterial infection and sepsis [4, 12–14], while lymphocyte complexity (LY-X), lymphocyte fluorescence intensity (LY-Y), and lymphocyte fluorescence distribution width index (LY-WY) have been reported as independent predictors for dengue fever [10].

Several previous studies have investigated each of these parameters to evaluate bacterial or viral infection, but there is no study that combines all these parameters to distinguish acute febrile illness, including dengue infection, chikungunya infection, typhoid infection, and other bacterial infections. The aims of this study are to explore the profile of these extended white blood cell parameters in common causes of febrile illnesses and to evaluate their diagnostic power to differentiate the etiologies of acute febrile illnesses.

## 2. Materials and Methods

This study was a cross-sectional analytical study conducted between October 2022 and October 2023 at Siloam Hospitals Lippo Village, Banten, Indonesia, and was approved by the Ethics Committee of Faculty of Medicine, Pelita Harapan University (Approval No. 160/K-LKJ/ETIK/III/2024). Data for extended white blood cell parameters were taken at the day of hospital admission. Acute febrile illnesses included in this study were dengue infection, identified by positive dengue NS1 or IgM rapid test, chikungunya infection by positive chikungunya IgM rapid test, typhoid infection by Tubex score > 4, and other bacterial infections by positive microbiological culture. Healthy subjects were taken from medical check-up samples without fever and normal laboratory result. Extended white blood cell parameters were included in full blood count as research parameters, performed by Sysmex XN2000. The analyzer was calibrated and maintained following the manufacturer's recommendation.

Data were analyzed using SPSS statistical software Version 25. The Shapiro–Wilk and Kolmogorov–Smirnov tests showed that the parameters had a skewed distribution, and the data were presented as median and interquartile range. A nonparametrical test, Mann–Whitney test, was performed to compare between two different groups, and the Kruskal–Wallis test was performed to compare three or more different groups, with *p* < 0.05 is considered as statistically significant difference and followed by paired comparison (Dunn's test) if significant. The area under the curve (AUC), sensitivity, and specificity were calculated.

## 3. Results and Discussion

### 3.1. Demographic Data

This study analyzed a total of 473 blood samples, including 413 patients diagnosed with acute febrile illnesses and 60 healthy subjects, and their demographics are presented in Table 1. Of the participants, 239 (50.53%) were male and 234 (49.47%) were female, with median age of 33 (14.5–54) years. The participants were classified into healthy control group (60 samples), dengue infection group (168 samples), chikungunya infection group (25 samples), typhoid infection group (80 samples), and other bacterial infection group (140 samples). Additionally, among the dengue infection group, 95 (56.55%) were positive NS1 dengue and 73 (43.45%) were positive IgM dengue. Among the other bacterial infection group, 66 (47.14%) had bacteremia, 38 (27.14%) had urinary tract infection, and 36 (25.72%) had pneumonia.

**Figure 1:**
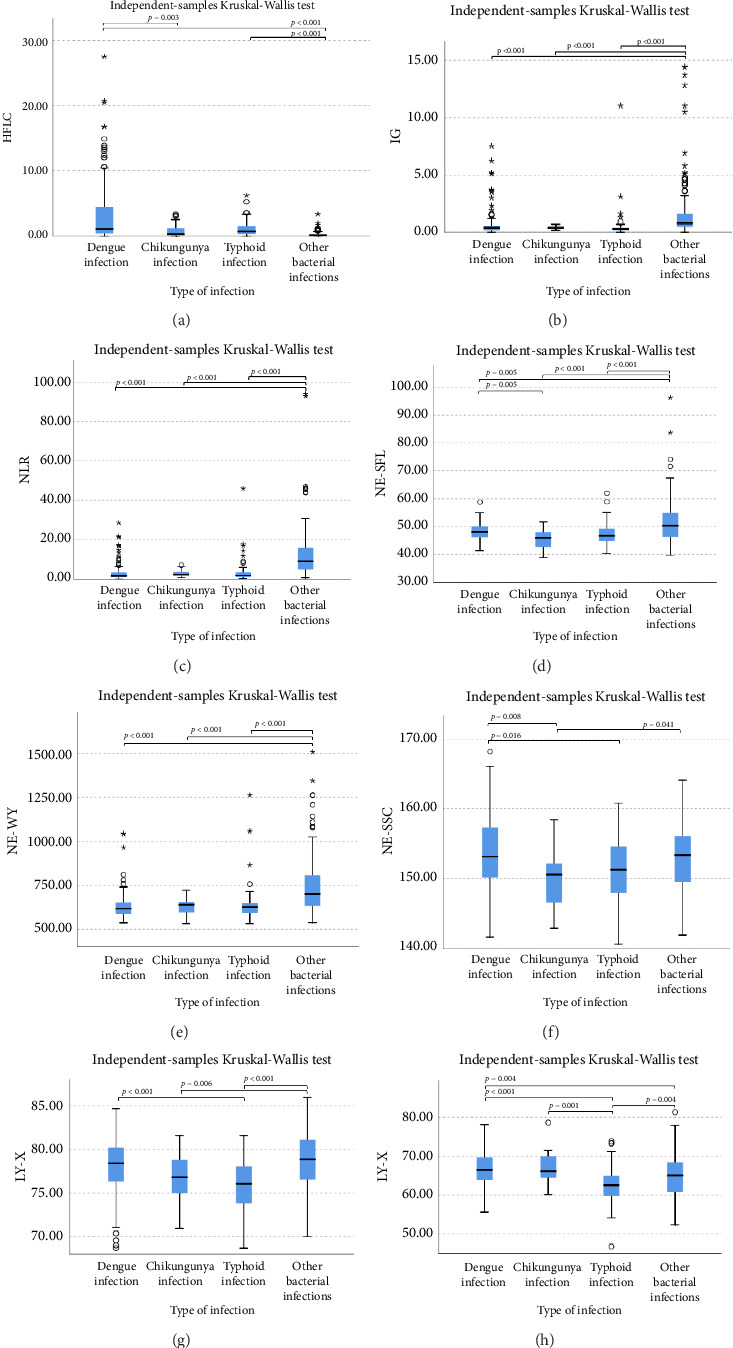
Comparison of extended white blood cell parameters and followed by Dunn's pairwise comparison, (a) HFLC, (b) IG, (c) NLR, (d) NE-SFL, (e) NE-WY, (f) NE-SSC, (g) LY-X, and (h) LY-Y, in acute febrile illnesses.

The extended white blood cell parameters for healthy subjects and acute febrile illnesses are displayed in Table 2. A Kruskal–Wallis test indicated that there were significant differences in extended white blood cell parameters across four acute febrile illnesses for HFLC, IG, NLR, NE-SFL, NE-WY, LY-X, LY-Y (*p* < 0.001), and NE-SSC (*p*=0.001). Post hoc comparisons using Dunn's method with a Bonferroni correction for multiple tests can be seen in Figure 1.

Pairwise comparison using Dunn's test indicated that HFLC was observed to be significantly higher in dengue infection from chikungunya infection and other bacterial infections (*p* < 0.001). NE-SSC was significantly lower in chikungunya infection compared to dengue infection (*p*=0.008) and other bacterial infections (*p*=0.041). LY-Y and LY-X were observed to be significantly lower in typhoid infection from dengue infection (*p* < 0.001), other bacterial infections (*p*=0.004 and *p* < 0.001, respectively), and chikungunya infection with *p*=0.001 for LY-Y, meanwhile IG, NLR, NE-SFL, and NE-WY were observed to be significantly higher in other bacterial infections compared to dengue infection, chikungunya infection, and typhoid infection (*p* < 0.001), Figure 1.

We also analyzed extended white blood cell parameters between the viral infection group (dengue and chikungunya infection) and the bacterial infection group (typhoid and other bacterial infections) by the Mann–Whitney test and found significantly higher in the viral infection group than bacterial infection group for HFLC 1.10% (0.30%–3.85%) vs. 0.20% (0.10%–0.70%), *p* < 0.001 and LY-Y 66.4 (63.85–69.75) vs. 64.05 (60.52–67.17), *p* < 0.001, meanwhile IG, NLR, NE-SFL, and NE-WY were higher in the bacterial infection group than in the viral infection group (0.5% [0.3%–1.1%] vs. 0.4% [0.2%–0.6%, *p* < 0.001; 5.21 [2.20–12.26] vs. 1.93 [1.10–3.47], *p* < 0.001; 48.6 [45.82–52.57] vs. 47.7 [45.95–50.10, *p*=0.020; and 653 (615–747) vs. 622 (585–653), *p* < 0.001; respectively).

### 3.2. Association of Extended White Blood Cell Parameters in Viral Infection

**Figure 2:**
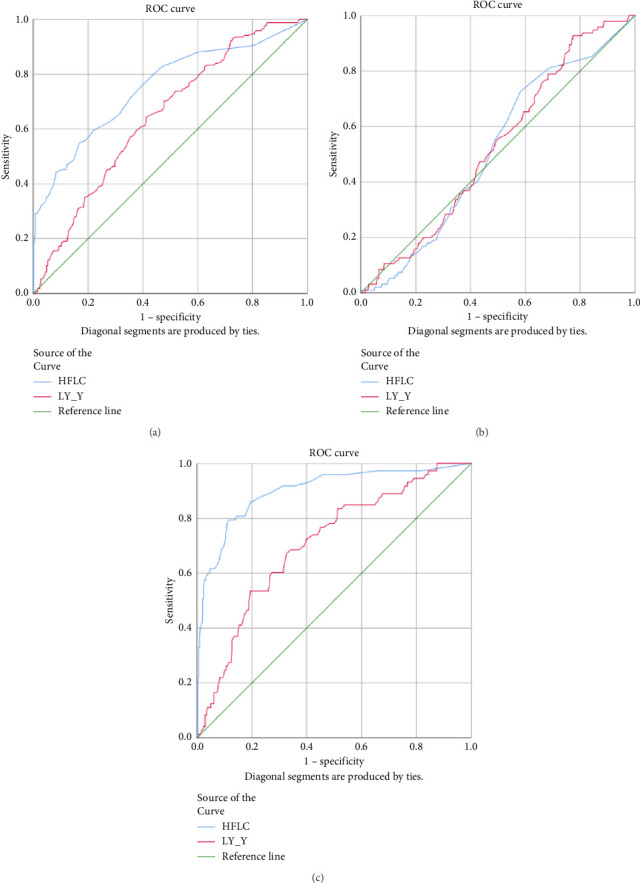
Receiver operating curve (ROC) for HFLC and LY-Y in predicting dengue infection group from other acute febrile illnesses (a), positive NS1 dengue group (b), and positive IgM dengue group (c).

HFLC and LY-Y had statistically significant AUC 0.753 and 0.646, respectively, (*p* < 0.001) in the dengue infection group compared to other acute febrile illnesses (Figure 2). Accordingly, a cut-off of HFLC 1.45% and LY-Y 65.15 was able to differentiate dengue from other febrile illnesses with sensitivity of 47.0% and 70.2% and specificity of 87.8% and 58.8%, respectively. We further analyzed HFLC and LY-Y across the positive NS1 dengue group and positive IgM dengue group and found that HFLC was significantly higher in positive IgM dengue. The AUCs of HFLC and LY-Y were 0.899 and 0.707 (*p* < 0.001) in the positive IgM dengue group and 0.516 (*p*=0.637) and 0.529 (*p*=0.392) in positive NS1 dengue infection (Figure 2). A cut-off of HFLC 1.95% and LY-Y 65.15 had higher sensitivity of 75.3% and 76.7% and specificity of 89.4% and 55% in the positive IgM dengue group, respectively. Meanwhile, the lower level of NE-SSC was associated with chikungunya infection, and the AUC was 0.665 (*p*=0.006).

### 3.3. Association of Extended White Blood Cell Parameters in Bacterial Infection

**Figure 3:**
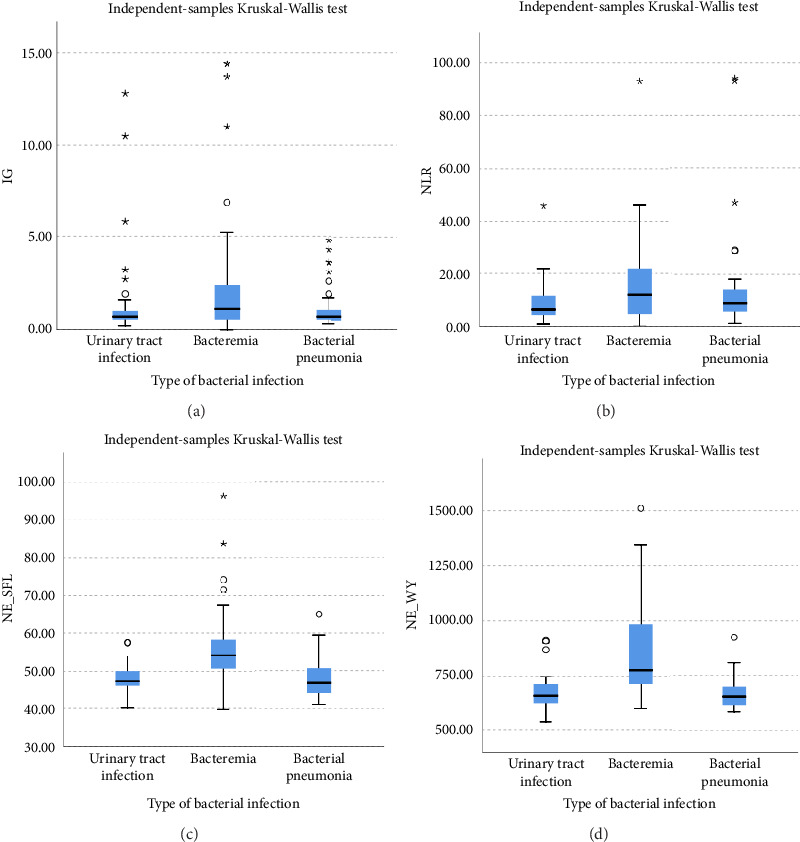
Box and whiskers plot of (a) IG, (b) NLR, (c) NE-SFL, and (d) NE-WY in type of other bacterial infections.

**Figure 4:**
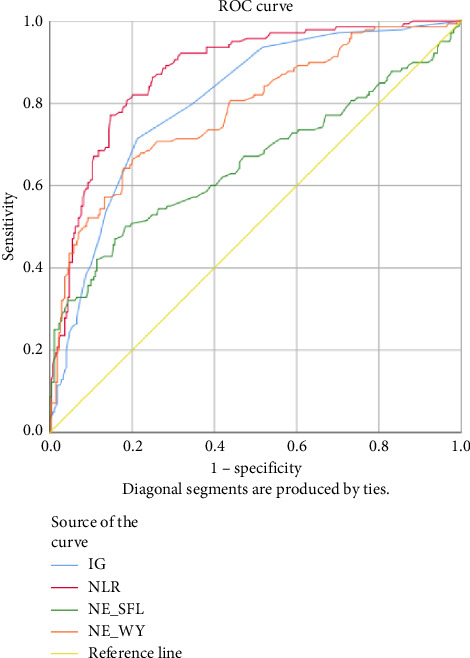
Receiver operating curve (ROC) of NLR, IG, NE-WY, and NE-SFL in other bacterial infections group compared to other acute febrile illnesses.

Lower levels of LY-X and LY-Y were associated with typhoid infection (*p* < 0.001) with AUC 0.714, sensitivity 76.3%, specificity 51.2%, cut-off value 62.6 and AUC 0.700, sensitivity 77.5%, specificity 51.2%, and cut-off value 76.15, respectively. Meanwhile IG, NLR, NE-SFL, and NE-WY were higher in other bacterial infections group rather than other acute febrile illnesses (Figure 1), particularly in the bacteremia group (Figure 3). Sensitivity, specificity, and cut-off value for these parameters were analyzed based on Youden's index. The AUC of NLR was 0.876 (sensitivity 90%, specificity 70%, and cut-off value 2.95), IG was 0.806 (sensitivity 71.4%, specificity 78.8%, and cut-off value 0.55), NE-WY was 0.783 (sensitivity 80.7%, specificity 56%, and cut-off value 627.5), and NE-SFL was 0.656 (sensitivity 50%, specificity 81.7%, and cut-off value 50.35), Figure 4.

## 4. Discussion

An automated hematology analyzer provides advanced technology to explore more information about the characteristics of white blood cell parameters, including HFLC, IG, NLR, and CPD. CPD provided information representing the volume, granularity, and complexity of each type of leukocyte, thus contributing to a more detailed study of the morphology in response to infection [4]. Since our study explored extended white blood cell parameters to distinguish viral and bacterial infection, we focused on the CPD of neutrophils (NE-SSC, NE-SFL, and NE-WY) for bacterial infection and lymphocytes (LY-WY, LY-X, and LY-Y) for viral infection.

Our study demonstrated that HFLC and LY-Y were higher in the viral infection group compared to the bacterial infection group. HFLC was an indicator of circulating plasma cells and antibody-synthesizing B-cells [15] and significantly correlated with atypical lymphocytes. Increased LY-Y is in proportion to increased cellular content of DNA and RNA and can be found in activated lymphocytes [16, 17]. Viral infection, particularly dengue infection, is known to have increased numbers of atypical lymphocytes and HFLC [15, 18, 19]. Chhabra et al. demonstrated that the dengue-positive cases showed a significantly increased HFLC among the different groups of controls, and HFLC at a cut-off value of 1.75% can discriminate between dengue-positive and dengue-negative patients with 52% sensitivity and 90% specificity [10]. Another study by Jayaram et al. stated that HFLC in cases and controls showed that at a cut-off value of 1.35%, HFLC reached optimal sensitivity and specificity of 82.8% and 87% [20]. Our study found that at a cut-off value of 1.45%, HFLC was able to differentiate dengue from other febrile illnesses with sensitivity and specificity of 47.0% and 87.8%, but if we analyze HFLC in the positive NS1 and IgM dengue groups separately, HFLC was not statistically significant in the positive NS1 group but had higher sensitivity and specificity in the positive IgM dengue group. This finding aligned with the previous study that stated HFLC was an indicator of antibody-synthesizing lymphocytes [15], and as IgM dengue was detected starting 4-5 days after the onset of symptoms [21], HFLC had not increased in the positive NS1 dengue group. Despite atypical lymphocytes and HFLC having a good correlation with viral infection, previous studies stated that there was no significant difference for atypical lymphocytes in chikungunya infection [22, 23].

For the bacterial infection group, IG, NLR, NE-SFL, and NE-WY were higher in our study compared to viral infection. IG is a precursor of white blood cells, including metamyelocytes, myelocytes, and promyelocytes, and previous studies demonstrated that IG can be a potential marker for bacteremia [6, 7, 24, 25]. Gungor et al. reported that IG had high sensitivity and specificity (75.4% and 76.6%, respectively) in predicting severe bacterial infection at a cut-off value of 0.35% [7]. Pavare et al. reported that a cut-off value of 0.45% had the best sensitivity and specificity for IG in children [26]. Our study found that at a cut-off value of 0.55%, IG had a sensitivity of 71.4% and a specificity of 78.8% to distinguish a bacterial infection from other infections.

NLR is a ratio of absolute neutrophil and lymphocyte count, with a higher result associated with bacterial rather than virus infection [8, 9, 27]. Meta-analysis of patient level data (five studies, *n* = 3320, AUC = 0.72) identified a cut-off value of 12.65 (sensitivity 66% and specificity 68%) for bacteremia, whereas bacterial respiratory and urinary tract infections had lower cut-offs (10 and 2.5, respectively) [27]. A previous study by Ng et al. stated that NLR reflected physiological stress when critically ill, regardless of microbiological etiology [28]. Our study combined three types of bacterial infection, which were bacteremia, respiratory tract infection, and urinary tract infection, and we found that at a cut-off value of 2.95, NLR had a sensitivity of 90% and a specificity of 70% to differentiate bacterial infection from other acute febrile illnesses.

Our study reported that NE-SFL and NE-WY had better specificity for other bacterial infections, with NE-WY having a better AUC than NE-SFL (0.783 vs. 0.656, respectively). This finding was in accordance with a study by Miyajima et al. that stated NE-WY had a stronger association with severity or sepsis markers than NE-SFL [12]. NE-SFL increases in proportion to the amount of cellular DNA or RNA, while increased NE-WY reflects the heterogeneity of the neutrophil population [12, 29]. In bacteremia, juvenile leukocytes with high nucleic acid content increase in peripheral blood, hence increasing the NE-SFL value. Despite the proliferation of juvenile leukocytes, bacterial infection induces neutrophil cell death as well, leading to varied nucleic acid content detected in peripheral blood and a high NE-WY [12].

Our study demonstrated that in chikungunya infection, lower level of NE-SSC as an indicator of neutrophil cell complexity, was statistically significant compared to dengue infection and other bacterial infections but did not have good diagnostic power since neutrophil activation and degranulation can be stimulated both in bacterial and viral infections [30]. Meanwhile, in typhoid infection, LY-X and LY-Y were observed to be significantly lower than other acute febrile illnesses because typhoid infection–induced lymphopenia by lymphocyte redistribution and margination within the lymphatic system [31]. However, LY-X and LY-Y had low diagnostic power for typhoid infection since there were overlapping distributions of these parameters in acute febrile illnesses, Figure 1.

This study had several limitations. First, a single-center and retrospective cross-sectional study may have resulted in selection bias and heterogeneity of disease status. Second, despite the overall total number of samples, this study had relatively small number of samples in the chikungunya infection group.

## 5. Conclusions

In this study, we demonstrated that extended white blood cell parameters can be used to distinguish common cause of acute febrile illnesses. HFLC was a useful diagnostic tool to identify viral infection, particularly dengue infection, while IG, NLR, NE-SFL, and NE-WY can be useful to differentiate bacteremia from other acute febrile illnesses.

## Figures and Tables

**Table 1:** Demographic profile of participants.

Characteristic	Total (*n* = 473), (%)
Age (years)	33 (14.5–54)
Gender	
Male	239 (50.53)
Female	234 (49.47)
Diagnosis	
Dengue infection	168 (35.52)
Positive NS1 dengue	95 (56.55)
Positive IgM dengue	73 (43.45)
Typhoid infection	80 (16.92)
Chikungunya infection	25 (5.28)
Other bacterial infections	140 (29.60)
Bacteremia	66 (47.14)
Urinary tract infection	38 (27.14)
Pneumonia	36 (25.72)
Healthy control subjects	60 (12.68)

**Table 2:** Extended white blood cell parameters in patients with acute febrile illnesses and healthy control subjects.

Variable	Diagnosis					*p* value^∗^
	Healthy subjects (*n* = 60)	Dengue infection (*n* = 168)	Chikungunya infection (*n* = 25)	Typhoid infection (*n* = 80)	Other bacterial infections (*n* = 140)	
TWBC	6.43 (5.62–7.75)	3.91 (2.63–5.34)	7.43 (5.66–8.80)	6.83 (5.17–9.24)	13.98 (9.71–18.36)	< 0.001
NEUT	3.76 (3.10–4.73)	1.98 (1.31–3.00)	4.98 (2.95–5.76)	3.89 (2.60–6.03)	11.34 (7.41–14.72)	< 0.001
LYMPH	1.99 (1.66–2.19)	0.92 (0.64–1.78)	1.73 (1.05–2.09)	1.93 (1.31–2.82)	1.12 (0.76–2.00)	< 0.001
HFLC	0.1 (0–0.2)	1.1 (0.4–4.4)	0.3 (0.05–1.85)	0.7 (0.3–1.57)	0.1 (0–0.3)	< 0.001
IG	0.3 (0.2–0.3)	0.4 (0.2–0.6)	0.4 (0.3–0.5)	0.3 (0.2–0.4)	0.8 (0.5–1.6)	< 0.001
NLR	1.91 (1.61–2.21)	1.8 (1.0–3.4)	2.26 (1.72–4.27)	1.94 (1–3.55)	8.89 (4.9–16.25)	< 0.001
NE-SFL	42.65 (41.72–44.95)	48.0 (46.1–50.1)	45.9 (42.6–48)	46.7 (44.75–49.2)	50.3 (46.2–55.07)	< 0.001
NE-WY	618.5 (594.8–635.5)	616 (584–652)	638 (590–655)	625.5 (591.5–649)	700.5 (632–807)	< 0.001
NE-SSC	149 (147–152)	153 (150–157)	150 (146.6–152)	151 (148–154)	153 (149–156)	0.001
LY-WY	861 (815–904)	905 (824–1014)	910 (852–964)	934 (852–1013)	929 (848–1028)	0.477
LY-X	76.4 (74.4–77.3)	78.4 (76.3–80.2)	76.8 (74.3–78.8)	76.05 (73.8–78.1)	78.9 (76.5–81.2)	< 0.001
LY-Y	63.7 (61.9–65.1)	66.4 (63.7–69.6)	66.1 (64.3–70.2)	62.45 (59.7–65)	65 (60.7–68.47)	< 0.001

*Note:* TWBC, total white blood cell count (10^9^/L); NEUT, neutrophil count (10^9^/L); LYMPH, lymphocyte count (10^9^/L); NE-SFL, neutrophil fluorescence intensity; NE-WY, neutrophil fluorescence distribution width index; NE-SSC, neutrophil cell complexity; LY-WY, lymphocyte fluorescence distribution width; LY-X, lymphocyte cell complexity; LY-Y, lymphocyte fluorescence intensity.

Abbreviations: HFLC = high fluorescence lymphocyte count (%); IG = immature granulocyte (%); NLR = neutrophil-to-lymphocyte ratio.

^∗^ *p* value is reported for comparison among acute febrile illnesses groups in Kruskal–Wallis.

## Data Availability

The dataset used to support the findings of the study is available from the corresponding author upon reasonable request.
